# Supplementary material for: Efficient, Breathable, and Compostable Multilayer Air Filter Material Prepared from Plant-Derived Biopolymers
Source: Membranes (Basel). 2023 Mar 27;13(4):380. doi: 10.3390/membranes13040380 (PMC10146039; doi:10.3390/membranes13040380)
Supplement: Supplementary file 1 [file membranes-13-00380-s001.zip › membranes-2253946-supplementary.pdf]

membranes

Supplementary Information

Efficient, Breathable, and Compostable Multilayer Air Filter Material Prepared from Plant-Derived Biopolymers

Rong Wu [1,†], Sneha Shanbhag [1,†] and P. Ravi Selvaganapathy [1,2,*]

[1] Department of Mechanical Engineering, McMaster University, 1280 Main St W, Hamilton, ON, L8S 4L8, Canada

[2] School of Biomedical Engineering, McMaster University, 1280 Main St W, Hamilton, ON, L8S 4L8, Canada

* Correspondence: selvaga@mcmaster.ca

† These authors contributed equally to this work.

Supplementary background

In the context of air filters, especially for facemask applications, standards and regulations such as ASTM F2100 (medical face masks or surgical masks), ASTM F3502 (barrier face coverings),[1] or regulations such as 42 CFR Part 84 (NIOSH N95 respirators)[2] provide testing guidelines and performance criteria. These tests characterize the filter materials' particle filtration efficiency and pressure drop under instantaneous conditions, high salt loading and high humidity. The pressure drop and particle filtration efficiency requirements for level 1 and level 2 barrier face covering and N95 respirators is provided in table S1.

Table S1. Summary of standards applicable to face coverings or face masks and respirators.

Standard	Mask type	Class (if applicable)	Testing air flow velocity	Particulate Filtration Efficiency (%)	Differential Pressure (Pa)
ASTM F3502	Barrier face covering	Level 1	10 cm/s	20%	147.1
	Barrier face covering	Level 2	10 cm/s	50%	49.03
NIOSH (42 CFR Part 84)	Respirator	Not applicable	85 l/min for a flat sample of 100 cm^2; 14.2 cm/s for respirator	>95% @ 0.075 +/- 0.2 μm count median diameter	343 Pa (inhalation); 245 Pa (exhalation)

Supplementary figures

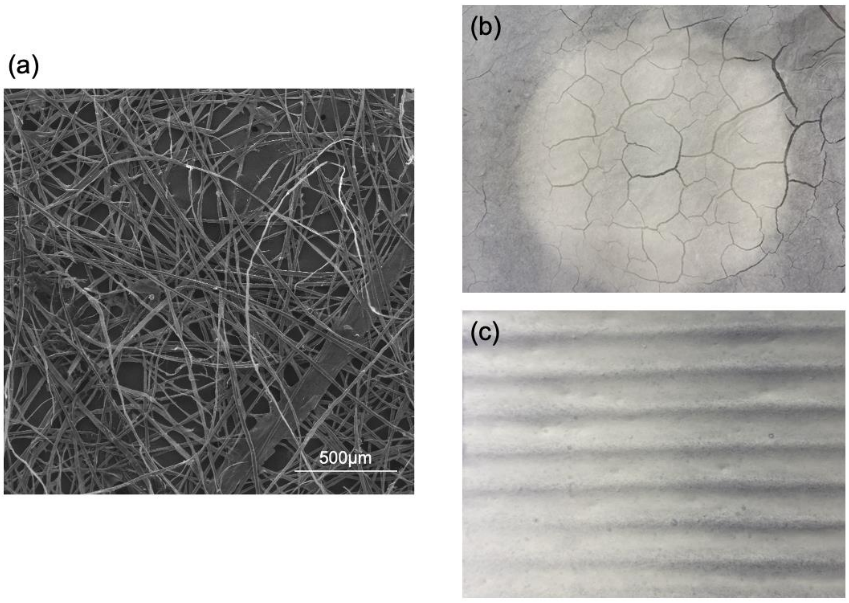

Figure S1. (a) SEM figure of crafted tissue paper substrate (b)Cracks on uncrosslinked zein filter after humidity conditioning for 1 hour; (c) un-uniform coverage of pleated filter along the fold lines.

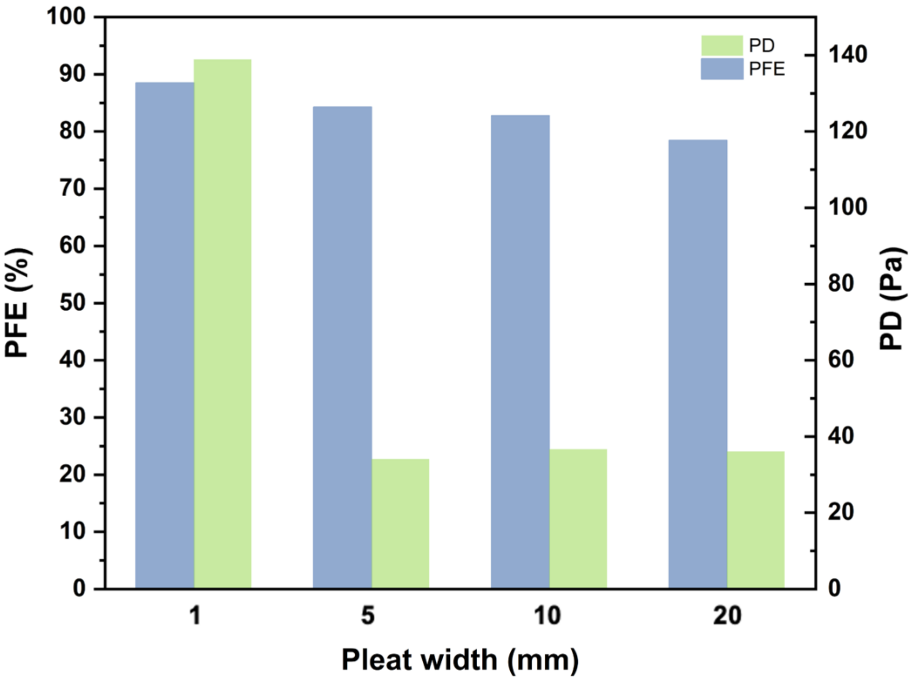

Figure S2. PFE and PD performance with different pleat width.

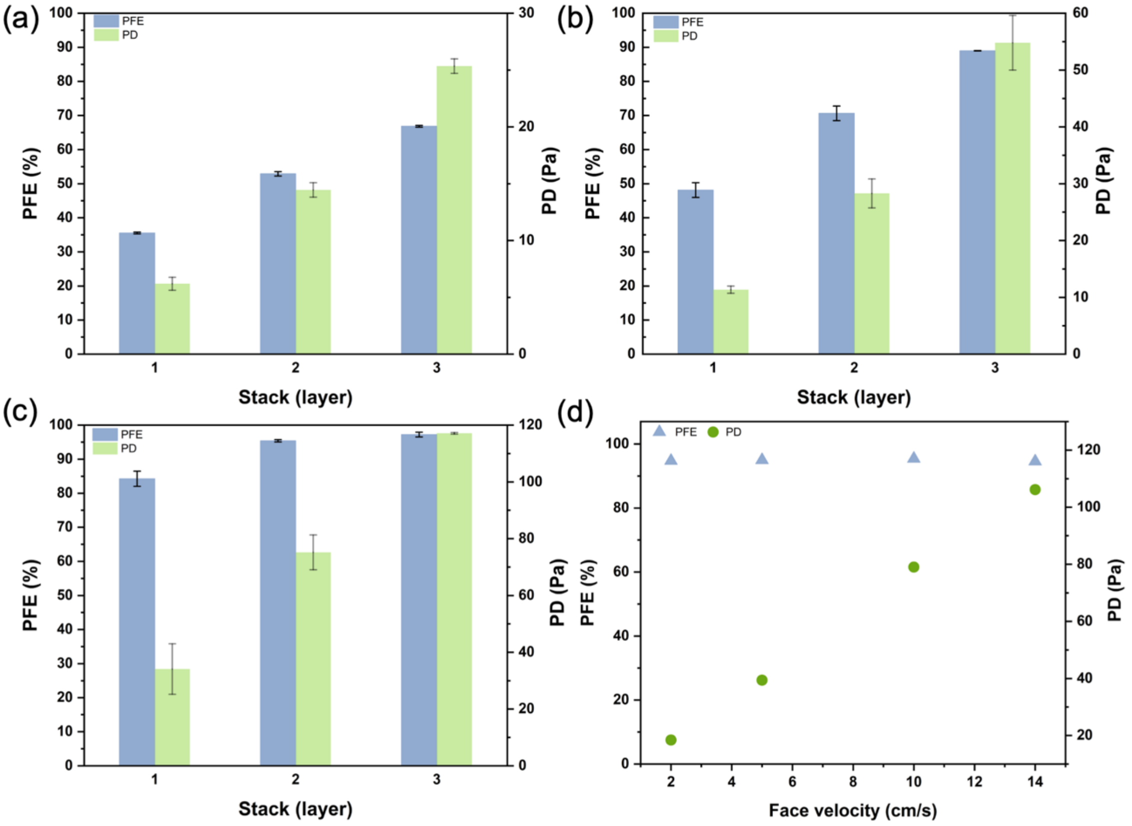

Figure S3. PFE and PD of pleated crosslinked zein filter at (a) 5 min; (b) 15 min; (c) 25 min, (d) PFE and PD of 2-layer stack of pleated zein filter at various face velocities.

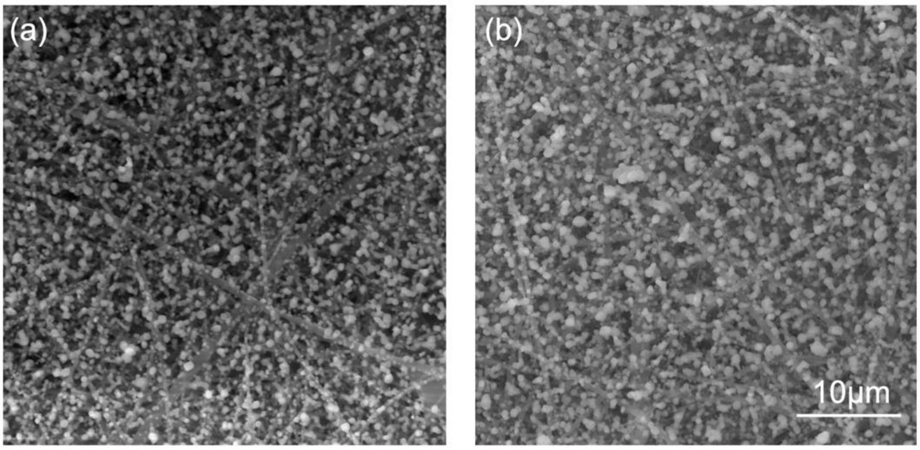

Figure S4. SEM images of (a) flat zein filter, (b) pre-pleated zein filter, after one hour loading test.

We also characterized the craft paper substrate, uncrosslinked filter, crosslinked filter materials by FTIR. The FT-IR spectra of those samples were measured via a Fourier transform infrared spectrometer, Bruker Vertex 70 spectrometer (Bruker Ltd., Germany) from 4000 to 500 cm^{-1}. The crosslinked zein and uncrosslinked zein filter with paper substrate

provided similar response under FTIR. The peak at 1481, 1566, and 1633 matched with previous study indicated the similar compounds in both samples[3].

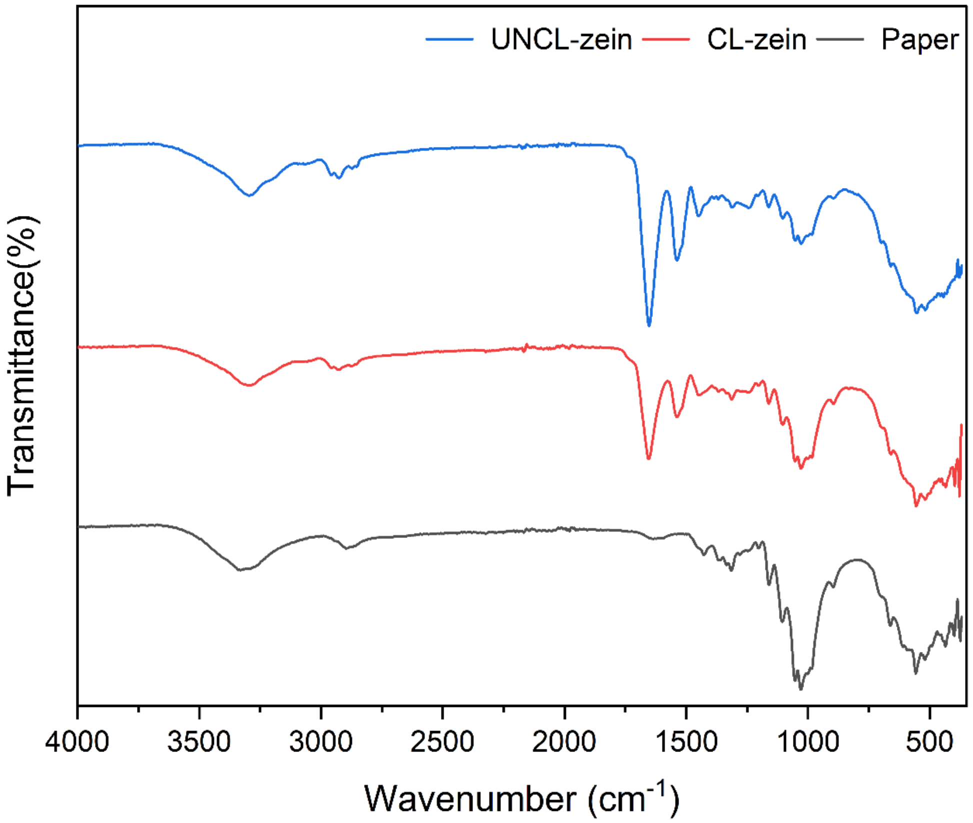

Figure S5. FTIR spectrum of paper substrate, crosslinked and uncrosslinked zein electrospun layer with paper substrate.

[1] *Standard Specification for Barrier Face Coverings*, A. S. f. T. a. Materials, February 15, 2021 2021.

[2] *Approval of respiratory protective devices*, T. N. I. f. O. S. a. Health, 1995.

[3] S. Ali, Z. Khatri, K. W. Oh, I.-S. Kim, and S. H. Kim, "Zein/cellulose acetate hybrid nanofibers: Electrospinning and characterization," *Macromolecular Research*, vol. 22, pp. 971-977, 2014.
